# Potential Viroporin Candidates From Pathogenic Viruses Using Bacteria-Based Bioassays

**DOI:** 10.3390/v11070632

**Published:** 2019-07-09

**Authors:** Prabhat Pratap Singh Tomar, Rivka Oren, Miriam Krugliak, Isaiah T. Arkin

**Affiliations:** Department of Biological Chemistry, The Alexander Silberman Institute of Life Sciences, The Hebrew University of Jerusalem, Edmond J. Safra Campus Givat-Ram, Jerusalem 91904, Israel

**Keywords:** viral channels, bacterial assays, channel blockers, anti-viral drugs

## Abstract

Viroporins are a family of small hydrophobic proteins found in many enveloped viruses that are capable of ion transport. Building upon the ability to inhibit influenza by blocking its archetypical M2 H^+^ channel, as a family, viroporins may represent a viable target to curb viral infectivity. To this end, using three bacterial assays we analyzed six small hydrophobic proteins from biomedically important viruses as potential viroporin candidates. Our results indicate that Eastern equine encephalitis virus 6k, West Nile virus MgM, Dengue virus 2k, Dengue virus P1, Variola virus gp170, and Variola virus gp151 proteins all exhibit channel activity in the bacterial assays, and as such may be considered viroporin candidates. It is clear that more studies, such as patch clamping, will be needed to characterize the ionic conductivities of these proteins. However, our approach presents a rapid procedure to analyze open reading frames in other viruses, yielding new viroporin candidates for future detailed investigation. Finally, if conductivity is proven vital to their cognate viruses, the bio-assays presented herein afford a simple approach to screen for new channel blockers.

## 1. Introduction

Many important viruses have been shown to contain small hydrophobic proteins that are capable of ion channel activity. Members of this family have therefore been collectively termed viroporins [1,2,3,4,5,6]. The best-characterized member of the viroporin family is the influenza M2 channel [7,8]. The protein garnered significant interest when it was shown to be the target of the aminoadamantanes, anti-viral drugs [9]. The mode of action of amantadine and rimantadine was subsequently shown to be blockage of the H^+^ channel activity of M2 [10].

Structurally, Influenza A M2 is a single-pass membrane protein that homo-tetramerizes to form the active protein complex [11]. X-ray crystallography, solution NMR, and solid-state NMR studies were able to derive structures of the transmembrane four-helix bundle [12,13,14]. Finally, since other viroporins are thought to possess a single transmembrane stretch, they may adopt a helical bundle structure, akin to the structure of M2.

Other than M2 from Influenza A, there are other examples of viroporins that have been characterized to a varying extent, amongst which one might mention: BM2 from Influenza B virus [15,16], CM2 from Influenza C [17,18], vpu from HIV-1 [19,20,21], p7 from Hepatitis C virus [22,23], Delta peptide from Ebola virus [24], NS2B from Japanese encephalitis virus [25] and from Dengue Virus [26], NS3 from bluetongue virus [27], and SH protein from human respiratory syncytial virus [28,29]. Finally, it is important to note that the importance of the channel activity of some of the aforementioned viroporins has been questioned in relation to the protein’s role in viral infectivity [30].

Ion channels, in general, have long been used as excellent targets for pharmaceutical point intervention. Therefore, identification of ion channels in viruses led to the hope that channel blockers may prove beneficial as anti-viral agents, as is the case in Influenza’s M2 channel [10]. Taken together, it is of no surprise that efforts are being spent to develop additional channel blockers against M2 and other viroporins [31,32,33,34,35].

The above examples have prompted us to search for additional viroporin candidates. As targets, we searched a select set of biomedically important viruses. These viruses we selected based on their pathogenicity, and paucity of channel characterization. In particular, we decided to focus on Eastern equine encephalitis (EEE) virus, West Nile virus, Dengue virus, and Variola virus. Scanning viral genomes and employing bacteria-based assays enabled us to identify six small proteins as potential viroporins.

EEE virus is a member of the Alphavirus genus, which is part of the Togaviridae, a family of positive-sense single-stranded RNA viruses. Other members of Togaviridae include Semliki Forest viruses, Ross River virus, Chikungunya virus, Sindbis virus, Rubella virus, and Barmah Forest virus. As a mosquito-transmitted disease, Eastern equine encephalitis impacts a large number of different hosts, and in humans causes encephalitis with a considerable mortality rate (33%), and appreciable brain damage amongst the survivors [36].

The West Nile virus is the etiological agent of the mosquito-borne West Nile fever, which has been reported all over the world, and against which there is currently no vaccine or medication [37]. Mortality rates among infected individuals in which the nervous system has been impacted are around 10%. In terms of taxonomy, the West Nile virus is a member of the Flavivirus genus within the Flaviviridae positive-sense single-stranded RNA viruses. The family contains other pathogens of biomedical importance, such as Dengue virus (see below), Yellow fever virus, and Zika virus.

The Dengue virus has represented an important health threat in recent times. It has been estimated that more than a third of the world population is located in regions in which Dengue fever is prevalent. The number of worldwide infections approaches 100 million annually, of which 500,000 results in dengue hemorrhagic fever and 22,000 in death [37].

Throughout the ages, smallpox has been one of the deadliest diseases known to man. In what has been heralded as a victory of modern medicine, it was completely eradicated in 1977, yet research continues since the virus remains a biological weapon threat. Variola virus, the etiological agent of smallpox, is a member of the Orthopoxvirus genus, which is part of the Poxviridae family of double-stranded DNA viruses [38].

## 2. Materials and Methods

### 2.1. Protein Search

Protein sequences from Eastern equine encephalitis virus, West Nile virus, Dengue virus, and Variola virus were extracted from the NCBI database. Subsequently, potential viroporins were identified using Phobius—a combined transmembrane topology and signal peptide predictor [39,40]. The method is based on a hidden Markov model that examines different regions within the signal peptide and the transmembrane segments. Transmembrane proteins shorter than one hundred amino acids were chosen for further analyses, as listed in Table 1. Additional transmembrane segment prediction algorithms were subsequently employed on the hits obtained from Phobius (TMpred [41] and MEMSAT [42]).

### 2.2. Bacterial Strains

Four strains of K12 *Escherichia coli* were used in the current study: DH10B, LB650, NT326, and LR1. DH10B cells were purchased from Invitrogen (Carlsbad, CA). LB650 bacteria (Δ*trkG*, Δ*trkH*, and Δ*kdpABC5* system) contain deletions in genes connected to potassium uptake [43] and were a kind gift from Professor K. Jung (Ludwig-Maximilians Universität München) and Professor G.A. Berkowitz (University of Connecticut). NT326 bacteria (Δ*malE*) lack an endogenous maltose binding protein (MBP) and were a kind gift from Professor D.M. Engelman (Yale University). LR1 bacteria contained a chromosomal copy of a pH-sensitive green fluorescence protein (GFP) called pHluorin [44] and were a kind gift from Professor M. Willemoës and Professor K. Lindorff-Larsen (University of Copenhagen).

### 2.3. Plasmids

All proteins were expressed as fusion proteins to the maltose binding protein using the pMAL-p2X plasmid (New England Biolabs, Ipswich, MA). Genes for corresponding proteins have been added with a nucleotide sequence coding for linker of seven amino acids, six histidines, and a stop codon at the 3′ end. EcoRI and XbaI restriction sites were located at the 5′ and 3′ ends, respectively. The sequences were synthesized by GenScript (Piscataway, NJ). Protein expression was achieved by adding isopropyl *β*-d-1-thiogalactopyranoside (IPTG) to the growth media, as indicated.

### 2.4. Chemicals

IPTG was purchased from Biochemika-Fluka (Buchs, Switzerland). All other chemicals were purchased from Sigma-Aldrich laboratories (Rehovot, Israel).

### 2.5. Growth Media

Lysogeny Broth (LB) was used for all bacterial growth [45], unless noted otherwise. LBK was similar to LB expect that KCl replaces NaCl at 10 gr/L. All media contained ampicillin at 100 μg/mL.

### 2.6. Bacterial Growth

*Escherichia coli* DH10B bacteria bearing or lacking (as a reference) the viral chimera were grown overnight in LB at 37 °C. Thereafter, the growth culture was diluted and the bacteria were grown until their O.D._600_ reached 0.2. Subsequently, 50 μL of bacterial culture was dispensed into 96-well flat-bottomed plates (Nunc, Roskilde, Denmark) containing 50 μL of the different treatments. Unless stated otherwise, IPTG was added to the cells to final concentrations ranging from 0 to 100 μM. d-glucose was added to a concentration of 1%. Moreover, 96-well plates were incubated for 16 h at 37 °C in an Infinite 200 from the Tecan Group (Männedorf, Switzerland) at a constant high shaking rate. O.D._600_ readings were recorded every 15 min. For every measurement, duplicates or triplicates were conducted.

For the *Escherichia coli* LB650 bacteria, the same protocol was used, except that growth was done in LBK overnight. Subsequently, the bacteria were diluted and grown until their O.D._600_ reached 0.2, after which the media was replaced to LB and diluted twofold with the various treatments in each well. Unless stated otherwise, IPTG was added to the LB650 bacteria to a final concentration of 10 μM.

### 2.7. Maltose Complementation Assay

All selected viroporin chimera were transformed into NT326 bacteria. Transformed cells were cultured on M9 minimal medium plates containing 0.4% maltose as the only carbon source, 1% Bacto agar (Difco, MD), and 100 μM IPTG for three days at 37 °C. Transformed cells expressing MBP (unfused to any viral gene) were used as a control.

### 2.8. pHlux Assay

Transformed LR1 cells with different viroporin chimera were cultured overnight in LB media containing 1% glucose and 100 μM ampicillin. Secondary cultures were prepared by diluting the primary culture by 1:500 in LB media and allowing it to grow up to an O.D._600_ of 0.6–0.8. Viroporin synthesis was induced by the addition of 50 μM IPTG for one hour. Cultures without IPTG induction were used as control. Following one hour of induction, the O.D._600_ of all cells were measured, and after pelleting at 3500 g for 10 min, the bacteria were resuspended in McIlvaine buffer (200 mM Na_2_HPO_4_, 0.9%NaCl adjusted to pH 7.6 with 0.1 M citric acid, 0.9%Nacl) to an optical density of 0.25 at 600 nm. Subsequently, 200 μL of cell suspension were transferred with 30 μL of McIlvaine buffer to a 96-well plate. The plate included a row with only an assay buffer and cultures without induction. The fluorescence measurements were carried out in a microplate reader (Infinite F200 pro, Tecan). The plate reader was set with two pairs of bandpass filters: A 520 nm emission filter combined with 390 nm and 466 nm excitation filters. A liquid handling system (Tecan) was used to add 70 μL of 300 mM citric acid with 0.9% NaCl to the bacteria. The fluorescence emission of each well after the addition of acid was measured by alternate read out of the two filter pairs for 30 s. The ratio for the two differently excited emissions, $F=F_{390\;\mathrm{nm}}/F_{466\;\mathrm{nm}}$ was calculated and translated into proton concentration using the following equation according to [46,47]:

$$\left[H^{+}\right]=0.132\cdot F^{-1.75\cdot F^{0.51}}.$$

### 2.9. Experimental Controls

Two controls were used in order to gauge the activity of the viroporin chimera: The first control involved the unadulterated pMAL-p2X plasmid (New England Biolabs, Ipswich, MA, USA) in which MBP is not fused to any protein at its carboxy terminus. The second control was one in which MBP was fused at its carboxy terminus to a “generic” transmembrane domain. Specifically, we placed the transmembrane domain of human glycophorin A (residues Glu70—Leu98) containing two monomerizing mutations: Gly79Ile and Gly83Ile [48,49].

## 3. Results

### 3.1. Sequence Analysis

**Figure 1:**
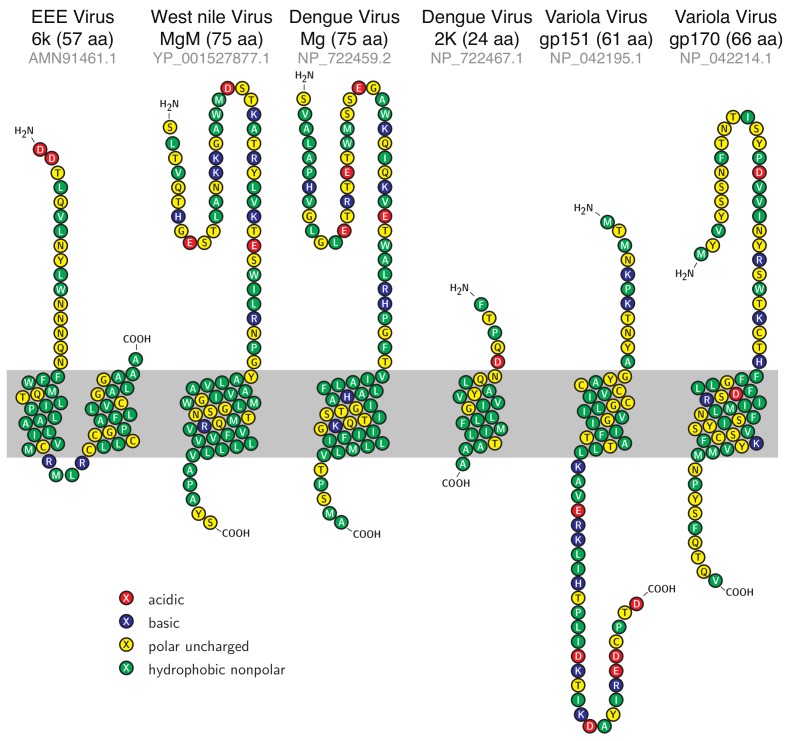
Sequence and topology of candidate viroporins. Sequences of the proteins analyzed in the current study alongside their predicted topologies according to Phobius [39,40]. The shaded region indicates the presumed position of the lipid bilayer. The lengths of the proteins are stated in the parentheses and the accession numbers in gray. The figure was prepared using T_E_Xtopo version 1.5 [56].

In order to identify potential viroporins, we utilized characteristic features of the viroporin family, as exemplified by the M2 proteins from influenza A, B, and C strains [10,17,50], and many other viruses [1,2,3,4,5,6]. Specifically, we searched for small (less than 100 residues) membrane proteins in genomes of the following enveloped viruses: Eastern equine encephalitis (EEE) virus, West Nile virus, Dengue virus, and Variola virus. These viruses represent important biomedical threats, and as such, any new drug target that is identified in their genomes may be of potential use. Using Phobius, a combined transmembrane topology and signal peptide predictor [39,40], we found six viroporin candidates listed in Table 1 and shown schematically in Figure 1 according to their predicted topology. Analyses employing TMpred [41] and MEMSAT [42] algorithms yielded similar results, with the exception of EEEV 6k protein, which was not identified as membranous by MEMSAT (see Table 2). Of the six proteins, EEE virus 6k is similar to homologous proteins from other alphaviruses that have been shown to possess ion channel activity, such as Sindbis virus [51], Ross River virus, Barmah Forest virus [52], and Semliki forest virus [53]. Similarly, a synthetic peptide that corresponds to part of the Dengue virus Mg protein has been shown to possess channel activity [54].

When comparing the sequences of the different putative viroporins, no appreciable similarities were detected. The only exception was between the two flaviviruses—West Nile virus MgM and Dengue virus Mg proteins (Table 3).

### 3.2. Expression and Membrane Incorporation

We next proceeded to express the proteins in several bacterial hosts in order to evaluate their ion channel activity. In an effort to ensure that the proteins are incorporated in the inner membrane of the bacteria, we constructed chimeras as follows: The amino-termini of the viral proteins were fused to the carboxy-terminus of the maltose binding protein (MBP) employing the pMAL™ Protein Fusion and Purification System from New England Biolabs. Note that MBP is monomeric, and as such does not induce oligomerization [57]. The secretion was subsequently directed by the signal peptide of MBP, placing the amino terminus of the viral protein in the bacterial periplasm. Similar constructs were shown to be successful with other viroporins such as Influenza M2 [58,59,60] and HIV vpu [61]. Finally, membrane incorporation could be confirmed using a genetic screen upon expression of the chimera in *mal*E^-^ bacteria. These bacteria are incapable of growing when maltose is the only carbon source since they lack a native MBP in the periplasm [62].

**Figure 2:**
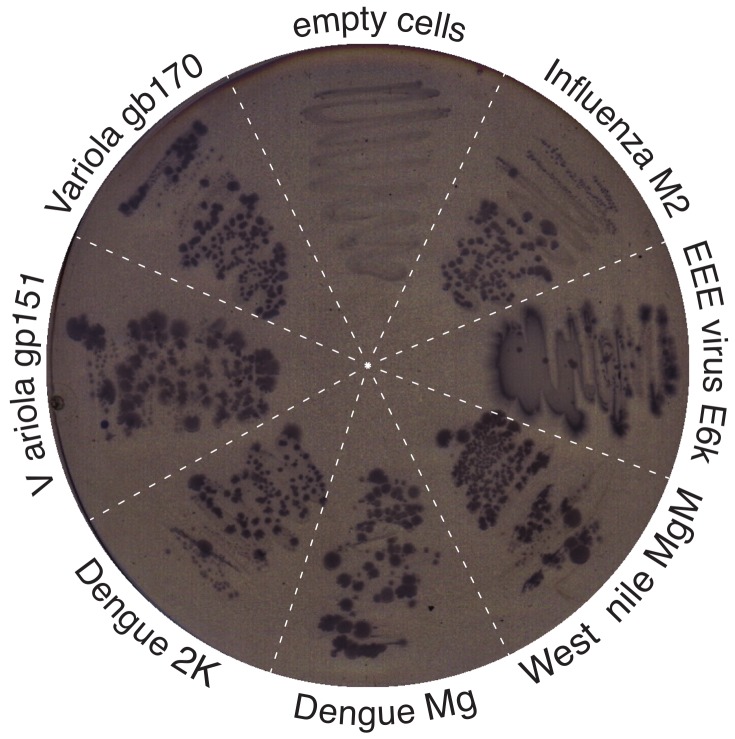
Membrane incorporation assay. Test for proper insertion and orientation of viral chimera based on the *malE*^-^ complementation assay [62]. Bacteria lacking any maltose binding protein (MBP) were transformed with various viroporins which were fused to MBP and cultivated on M9 agar containing 0.4% maltose and 10 μM isopropyl *β*-d-1-thiogalactopyranoside (IPTG).

As shown in Figure 2, the expression of all chimeras was able to sustain bacterial growth when maltose was the only carbon source. In contrast, bacteria that did not express an MBP chimera were nonviable under these conditions. In conclusion, these findings demonstrate that all the viral proteins were inserted in the periplasmic membrane of the bacteria such that their N-termini are in the periplasm.

### 3.3. Positive Genetic Test

We have previously developed a genetic selection capable of examining the ability of a particular protein to conduct K^+^ [60,61]. The assay is based on the inability of K^+^-uptake-deficient bacteria [43] to grow on media containing low concentrations of potassium. However, when the bacteria express a protein capable of K^+^ transport, they are suddenly able to thrive under low K^+^ conditions.

**Figure 3:**
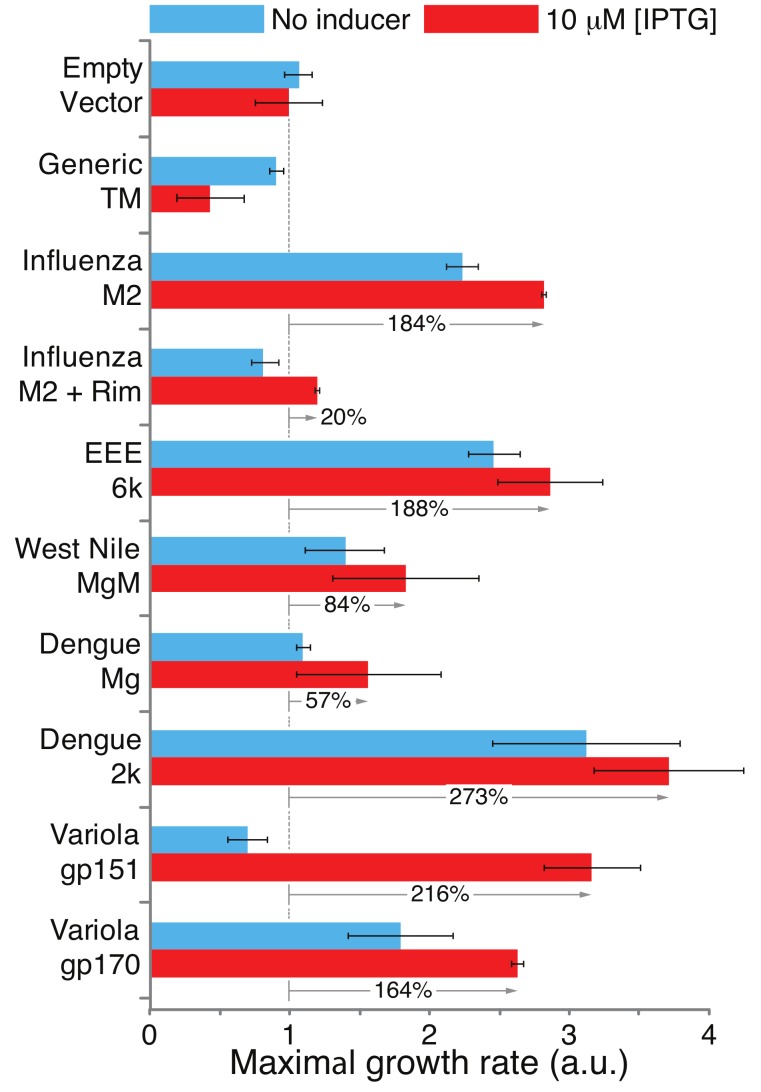
Positive genetic test for viroporin activity. Impact of viral protein expression on the growth of K^+^-uptake-deficient bacteria [43]. The relative growth enhancements in comparison to bacteria that harbor an empty vector (MBP on its own) are indicated by the gray arrows. Generic TM indicates a chimera in which MBP is fused at its carboxy terminus to the transmembrane domain of glycophorin A containing two monomerizing mutations [48,49]. M2 + Rim is a sample in which rimantadine, a specific blocker of the influenza M2 channel [10], was added at a concentration of 50 μM. Note that rimantadine did not affect other proteins (data not shown). The results are an average of three independent experiments, with standard deviations depicted as error bars.

Results shown in Figure 3 demonstrate appreciable growth enhancement in bacteria that harbor viral chimeras. For example, the expression of the Dengue virus 2K and Mg1 chimeras increases the growth rates of the bacteria by 273% and 57%, respectively. Furthermore, in several instances, increasing the expression of the chimeric proteins beyond basal levels (by adding the inducer IPTG), enhances the growth rate appreciably. In other instances, the basal level expression is sufficient to beneficially impact bacterial growth. As a control, bacteria that express MBP or MBP fused to a generic transmembrane domain do not exhibit appreciable growth enhancement. Taken together, all the viral proteins are capable of supporting K^+^ transport in the assay employed.

### 3.4. Negative Genetic Test

In complementary fashion to the positive genetic test detailed above, we have devised a genetic selection in which channel formation is detrimental to bacteria [58]. In this instance, the chimeric protein is expressed at elevated levels in “regular” *Escherichia coli*. This results in appreciable growth retardation, presumably due to extensive membrane permeabilization. This particular assay has the additional benefit that it may be used to search for blockers that mitigate the deleterious effect of the viral channel [59].

**Figure 4:**
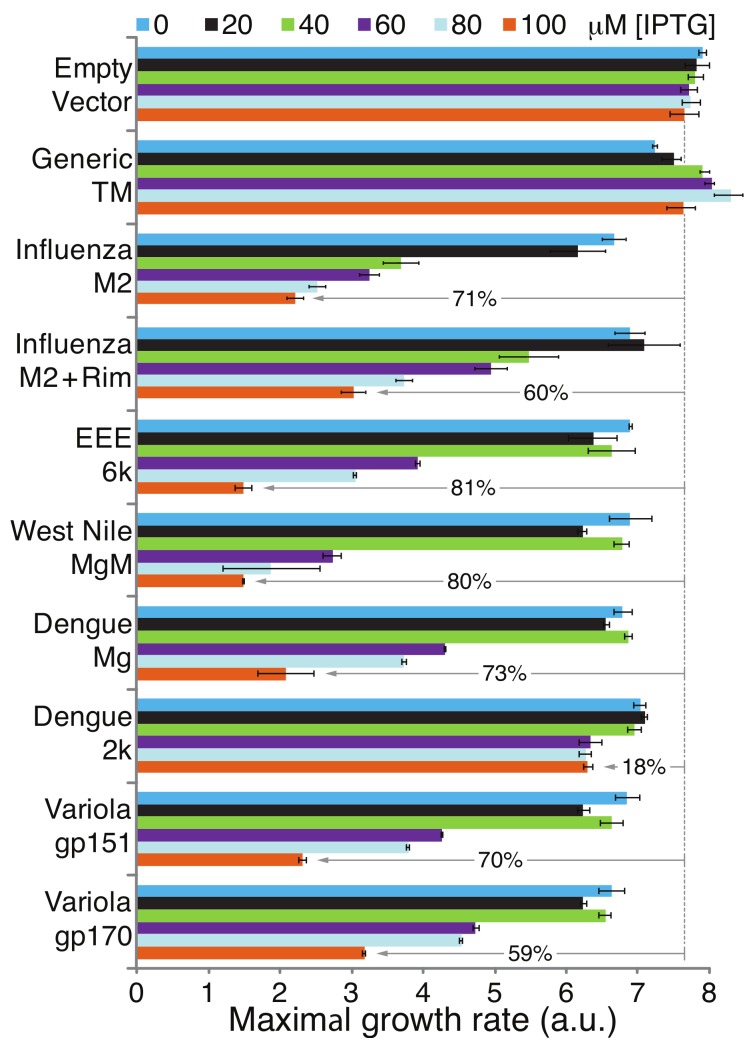
Negative genetic test for viroporin activity. The impact of viral protein expression on bacterial growth as a function of the concentration of the inducer IPTG varied between 0 to 100 μM, as indicated. The relative growth inhibitions in comparison to bacteria that harbor an empty vector (MBP on its own) are indicated by the gray arrows. Generic TM indicates a chimera in which MBP is fused at its carboxy terminus to the transmembrane domain of glycophorin A containing two monomerizing mutations [48,49]. M2 + Rim is a sample in which rimantadine, a specific blocker of the influenza M2 channel [10], was added at a concentration of 50 μM. Note that rimantadine did not affect other proteins (data not shown). The results are an average of three independent experiments, with standard deviations depicted as error bars.

Figure 4 depicts the results of the negative genetic assay of the various chimeras. The growth rates of bacteria that harbor various vectors are analyzed as a function of the concentration of the expression inducer (IPTG). Bacteria that harbor a vector that contains only MBP do not exhibit appreciable growth retardation when protein induction is increased. Similarly, bacteria that express a chimera in which a generic transmembrane domain is fused to MBP, do not exhibit any reduction in growth rates as a function of inducer concentration. In contrast, substantial growth impairment is observed when the bacteria express viral chimera. For example, increasing the IPTG concentration to 100 μM lowers the maximal growth rate by 81% in bacteria that express the Eastern equine encephalitis virus 6k protein. The only exception is bacteria that express the Dengue virus 2k protein, which exhibits only an 18% growth reduction.

### 3.5. Proton Flux

The final bacterial assay that was used measured H^+^ conductivity. Bacteria that constitutively express a pH-sensitive green fluorescent protein—pHluorin [44], can be used to analyze the membrane permeation to H^+^s [46,47]. Specifically, the emission at 520 nm of pHluorin has two excitation maxima: 390 nm and 466 nm, whose ratio changes as a function of pH [44]. Consequently, using a calibration curve enables one to relate the fluorescent quotient directly to the H^+^ concentration [46,47].

Experimentally, we monitored the fluorescence obtained when exciting at 390 nm and 466 nm upon injection of a concentrated acid solution to the buffer. Any bacteria that express an H^+^-conducting channel will exhibit a dramatic change in the fluorescence ratio in contrast to control bacteria that contain an empty vector.

**Figure 5:**
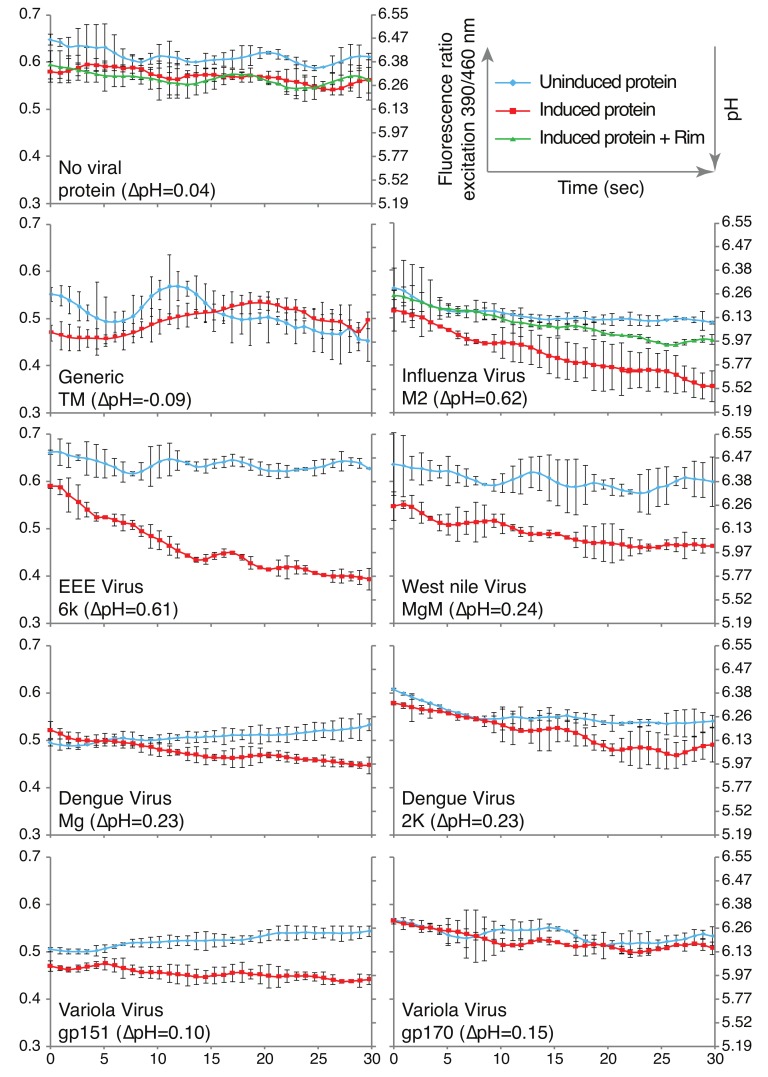
Fluorescence-based H^+^ conductivity assay. The fluorescence of bacteria that harbor pHluorin, a pH-sensitive green fluorescence protein (GFP) [44], was examined as a function of the expressed viral chimera [46,47]. Protein levels were governed by the level of the inducer (50 μM IPTG), as indicated. At time 0, a concentrated acid solution was injected into the media. Consequently, a drop of the fluorescence ratio between excitation at 390 nm relative to excitation at 466 nm (shown on the left axis) is indicative of a pH decrease, as shown on the right axis. The ΔpH values indicated in the parentheses correspond to the difference in the pH after 30 s in bacteria that had undergone protein induction. Generic TM indicates a chimera in which MBP is fused at its carboxy terminus to the transmembrane domain of glycophorin A containing two monomerizing mutations [48,49]. + Rim is a sample in which rimantadine, a specific blocker of the influenza M2 channel [10], was added at a concentration of 50 μM. Note that rimantadine did not affect other proteins (data not shown). The results are an average of three independent experiments, with standard deviations depicted as error bars.

As shown in Figure 5, bacteria that do not express a viral protein exhibited only a minor pH drop, from 6.31 to 6.27. Similarly, no drop in pH was observed in bacteria that express a chimera between MBP and a generic transmembrane domain. In contrast, as a positive control, bacteria that express the influenza virus M2 protein, a well-characterized H^+^ channel [10], exhibited a substantial increase in acidity of ΔpH=0.62.

All viral proteins exhibited acidity increases that were larger than the negative controls (i.e., MBP or MBP fused to a generic transmembrane domain), albeit at different levels. For example, the expression of Eastern equine encephalitis virus 6k protein resulted in a large acidity drop of ΔpH=0.61, while Variola virus gp151 resulted in only a small pH drop of 0.1 units. Finally, we note that minor differences in pH starting points may reflect experimental variability in growth conditions and potentially basal expression levels of the viral chimera.

## 4. Discussion

The goal of this study was to identify potential ion channels from biomedically important viruses. Scanning the genomes of Variola, Dengue, West Nile, and Eastern equine encephalitis viruses resulted in the identification of several candidate proteins that were less than one hundred amino acids in length and contained one or more putative transmembrane segments. The shortest open reading frame was Dengue virus 2K protein with 24 amino acids, while the longest was West Nile virus MgM and Dengue virus Mg1, each with 75 amino acids (see Figure 1). No similarities were observed between the different viroporin sequences, other than that observed between the two falvivirus proteins (Table 3). The reason for this finding might be that viroporins were not selected in the course of evolution to exhibit precise conductivity characteristics, as the channels in the neuromuscular system. Therefore, the sequence requirements for simple conductivity allow for great variation in sequence. Finally, as these features are all indicative of viroporins [1,2,3,4,5,6], we decided to examine the functionally of the proteins in three different bacterial assays.

Prior to any conductivity tests, it was important to ensure expression and membrane incorporation in bacteria. To that end, we constructed chimeric proteins in which the different viral proteins were attached to the carboxy terminus of the maltose binding protein (MBP). In such constructs, MBP, due to its leader peptide, would cause the chimera to be targeted to the periplasm causing the viroporin to traverse the inner bacterial membrane with its amino terminus in the periplasm. *mal*E^-^ complementation assay [62] did indeed reveal that all proteins were targeted to the inner membrane of the bacteria (Figure 2).

Following confirmation of membrane incorporation of the various proteins, we could analyze their potential conductivity using three independent assays. In the first assay, any protein that was capable of potassium transport could revive the growth of K^+^-uptake-deficient bacteria [43]. In the second assay, the chimeric proteins were expressed at varying levels in bacteria and their deleterious impact upon growth, due to excess membrane permeabilization, was monitored. The final test that was used measured H^+^ conductivity using a pH-sensitive green fluorescent protein called pHluorin [44].

All of these assays had already been tested before on the best-characterized viroporin—the M2 channel from Influenza A [46,47,58,59,60]. This particular channel has the advantage that it has an effective blocker that can negate its channel activity [10], thereby demonstrating the assays’ specificity. Therefore, the results of examining the M2 channel, in these three assays, in the presence or absence of its specific inhibitor, are presented as controls. Finally, two additional negative controls are utilized: MBP fused to a generic transmembrane domain and MBP on its own.

All of the viral proteins scored positively (above control) in the three bacterial assays, albeit at different levels. Since the different bacterial assays test non-identical characteristics of the proteins, they should be used in concert in order to predict channel activity. Hence, we may conclude that all of the aforementioned proteins may be classified as potential viroporin candidates, open for future detailed investigation. Moreover, the approach that we have taken can be readily employed to examine numerous other open reading frames, yielding additional targets for research.

Amongst future studies of the candidate proteins, one may mention the detailed characterization of the channel activity by approaches such as patch clamping. In addition, it is important to ascertain the relevance of the conductivity of these proteins to their cognate viruses. If their channel activities are shown to be essential for viral infectivity, the negative assay used in their identification (Figure 4) may be used to search for blockers. Such inhibitors could be classified as potential new anti-viral agents, as was done for other viroporins [59].

## Figures and Tables

**Table 1:** List of candidate viroporins analyzed in the current study. The sequences and topologies of the proteins according to Phobius [39,40] are shown in Figure 1. EEE is Eastern equine encephalitis.

Virus	Protein	Accession No.	Length
EEE virus	6k protein	AMN91461.1	57
West Nile virus	Membrane glycoprotein M (MgM)	YP_001527877.1	75
Dengue virus 1	Membrane glycoprotein (Mg)	NP_722459.2	75
Dengue virus 1	2k Protein	NP_722467.1	24
Variola virus	gp151	NP_042195.1	61
Variola virus	gp170	NP_042214.1	66

**Table 2:** Transmembrane segment prediction of the candidate viroporins analyzed in the current study. See Table 1 for complete names of proteins and accession numbers. All programs were run with default values.

Proteins	Phobious [39,40]	TMpred [41]	MEMSAT [42]
EEEV 6k protein	17–33; 38–56	16–37; 38–57	No TM detected
WNV MgM	42–70	42–75	41–58
DV Mg	44–70	41–75	44–68
DV 2k	6–23	8–24	Too short for prediction
VV gp151	12–32	12–32	12–32
VV gp170	30–57	39–57	39–57

**Table 3:** Pairwise comparisons (E-values) between the candidate viroporins analyzed in the current study. Comparisons were conducted with the blasptp algorithm [55], using default values, whereby the protein in the column was used as a reference. A “-” sign designates that the program was unable to find a significant similarity between the sequences, while a black cell indicates self-similarity. See Table 1 for complete names of proteins and accession numbers.

	6k	MgM	Mg	2k	gp151	gp170
6k		0.077	2.9	4.5	-	0.75
MgM	0.031		$3\times 10^{-10}$	0.32	-	-
Mg	2.9	$10^{-14}$		4.3	-	-
2k	-	-	-		-	-
gp151	-	-	-	-		-
gp170	0.75	-	-	0.003	-	

## Abbreviations

The following abbreviations are used in this manuscript:

EEE	Eastern equine encephalitis
MBP	Maltose binding protein
HIV	Human immunodeficiency virus
IPTG	isopropyl *β*-d-1-thiogalactopyranoside
LB	Lysogeny Broth
LBK	Lysogeny Broth in which Na^+^ is substituted by K^+^
TM	Transmembrane
